# Filaria in the Eye of a Dog

**Published:** 1896-04

**Authors:** 


FILARIA IN THE EYE OF A DOG. A six-year-old shepherd's dog had kerato-conjunctivitis in the right eye. As the dog bit the disease was neglected, so that panophthalmitis developed. When the animal was received by Rossi for treatment a fistula of the cornea was present, out of which a part of the iris had slipped forward. The front chamber of the eye was filled up with a purulent, fibrinous exudation.

The eye was extirpated. On cutting through the bulb there was found in the middle of the exudation a female filaria, whose species the writer cannot determine. It had a pale-red filiform body striped lengthwise, of about 14.7 cm. in length and about ½ mm. in diameter. The head-end was rounded off, the tail straight and pointed, the mouth circular and bare, the œsophagus short. A slight widening of the intestine formed the stomach, which was coiled. The anal opening lay 1 mm. from the end of the tail. The uterus was double, dangled about, and contained a small quantity of eggs with embryos. The vaginal opening was separated about 5 mm. from the mouth.—*Berliner thierärztl. Wochenschrift*, 1895, No. 47, in January number of *Tijdsch. voor Veeartsenijkunde.*

